# Botulinum toxin type A interrupts autophagic flux of submandibular gland

**DOI:** 10.1042/BSR20190035

**Published:** 2019-07-23

**Authors:** Shang Xie, Hui Xu, Xiao-Feng Shan, Zhi-Gang Cai

**Affiliations:** 1 Department of Oral and Maxillofacial Surgery, Peking University School and Hospital of Stomatology, National Engineering Laboratory for Digital and Material Technology of Stomatology, Beijing Key Laboratory of Digital Stomatology, 22# Zhongguancun South Avenue, Haidian District, Beijing 100081, China; 2 Department of General Dentistry, Wang Fujing Branch, School of Stomatology, Capital Medical University, 11# Xila Hutong, Dongcheng District, Beijing 100006, China

**Keywords:** autophagy flux, autophagy, botulinum toxin type A, LC-3, p62, submandibular gland

## Abstract

Botulinum toxin type A (BTXA) is a neurotoxic protein produced by *Clostridium botulinum*. Our previous studies demonstrated that BTXA inhibits the secretory function of submandibular gland (SMG) and changes its structure. Several studies reported that SMG damage and repair often occur with autophagy in the rat. However, no studies reported whether secretory inhibition and structural changes of SMG after BTXA injection is related with autophagy. The present study was carried out to explore the association between BTXA injection and autophagy in rat SMG. Western blotting and immunofluorescence were used to detect the expression and distribution of light chain 3 (LC3) in rat SMG. MTS was used to detect the toxicity of BTXA on rat SMG-C6 cell line. GFP-LC3 and Lyso-Tracker Red fluorescence probe were used to assess the levels of autophagosomes and lysosome fusion and the effect of BTXA on autophagic flux in SMG-C6. Western blotting and immunofluorescence results showed that BTXA temporarily increased autophagosomes in rat SMG. MTS results showed that BTXA exerted its toxicity on SMG-C6 in a dose-dependent manner. BTXA increased the number of autophagosomes in SMG-C6; however, most autophagosomes did not colocalize with lysosome. Therefore, we presume that BTXA can change autophagic flux of SMG cells, the mechanism of which might relate with BTXA’s disturbing autophagosome-lysosome fusion.

## Introduction

Botulinum toxin (BTX) is a neurotoxic protein produced by *Clostridium botulinum* [1–3]. Amongst the eight BTX types (A–H), Botulinum toxin type A (BTXA) has been widely used to treat excessive gland secretion, like hyperlacrimation, drooling, and Frey’s syndrome [4,5]. Our previous studies confirmed that BTXA can temporarily inhibit submandibular gland (SMG) secretion, a process that arose from BTXA-induced SMG cell apoptosis and aquaporin 5 (AQP5) expression and distribution [6–8]. However, the mechanism about how BTXA inhibits salivary secretion and changes the tissue structure of SMG remains unclear.

Autophagy plays in the development of eukaryotic cells and related diseases [9–11]. It has four stages, namely the formation of a separation membrane, the formation of autophagosomes, the fusion of autophagosomes and lysosomes, and the degradation of autophagic products. The activation of autophagy requires many proteins, amongst which light chain 3 (LC3) is essential for autophagosome formation, and SQSTM1/p62 (sequestosome 1, p62) for autophagic substrate degradation. Autophagic flux is a process by which phagosomes fuse into lysosomes and degrade their contents [12,13]. This process occurs not when autophagy is going, but when autophagy is completed [12]. Autophagy is currently considered to be closely related to tumors, cardiovascular diseases, neurodegenerative diseases, aging, autoimmune diseases, and tissue fibrosis [10,14–19].

In 2010, Silver et al. found that glandular injury repair was accompanied with activation of autophagy and mTOR signaling pathway [20]. Zoukhri et al. showed that IL-1β-induced repair of lacrimal gland inflammation was accompanied with autophagy in mice [21]. The above studies verified the association between glandular repair and autophagy. However, whether the inhibited salivary secretion and changed glandular structure after BTXA injection are related to autophagy remains unclear. To answer this question, the present study explored the associations between BTXA injection and autophagy and autophagic flux in rat SMG at tissue and cell levels.

## Materials and methods

### Experimental animals and BTXA injection

Healthy adult male SD rats (body weight 230–250 g) were purchased from the Experimental Animal Center of Peking University Health Science Center. All experimental procedures were approved by the Animal Research Ethics Committee (Peking University Health Science Center) and conformed to the Guide for the Care and Use of Laboratory Animals published by the US National Institutes of Health (NIH publication No. 85-23, revised 1996).

For BTXA injection, 18 rats were randomly divided into six groups (three per group), including control group and five experimental groups (1 week group, 2 weeks group, 4 weeks group, 12 weeks group, and 24 weeks group). Under anesthesia, the rats were placed in a supine position and routinely sterilized. A midline incision was made in the neck to expose the bilateral submandibular glands. Each rat in the control group was injected with 0.1 ml of normal saline in the left submandibular gland. In order to study the effect of BTXA on the submandibular gland, each rat in the BTXA group was injected with three units BTXA (dissolved in 0.1 ml of normal saline). The submandibular gland specimens were collected under anesthesia at 1, 2, 4, 12, and 24 weeks after BTXA injection.

### Reagents and antibodies

BTXA was gifted by Lanzhou Institute of Biological Products, China. One unit BTXA from this institute is equal to one unit of BOTOX (Allergan, Irvine, CA, U.S.A.) and 3–4 units of Dysport (Ipsen, Slough, U.K.). Antibodies for LC3-II and SQSTM1/p62 were purchased from Cell Signaling Technology (Beverly, MA, U.S.A.). *β*-actin antibody and FITC were purchased from Santa Cruz Biotechnology (Santa Cruz, CA, U.S.A.). CellTiter 96^®^AQueous One Solution cell proliferation assay (MTS assay) was purchased from Promega (Madison, WI, U.S.A.).

### Cell culture and transfection

The rat submandibular gland SMG-C6 cell line was cultured in DMEM/F12 (1:1) as previous description [7,22]. The cells were cultured in a 37°C incubator containing 5% CO_2_ and transfected with cDNA of green fluorescent protein (GFP)–LC3 by use of MegeTran 1.0 (OriGene) according to the manufacturer’s instructions. After 24 h, the cells were kept in culture medium with 200 μg/ml G418 and maintained for 5 d.

### Cell viability assay

The toxic effect of BTXA on SMG-C6 cell line was detected using MTS assay. The cells were seeded in 96-well plates (5000 cells per well, 100 μl per well), treated with various concentrations of BTXA (0, 50, 100, 200, and 300 U/ml), and maintained for 30 min, 24, 48, and 72 h, respectively. At each time point, 20 μl of MTS solution was added to each well and allowed to culture for 2–4 h. After incubating, the light absorption value of each well was measured at the wavelength of 490 nm on an enzyme-linked immunosorbent monitor.

### Immunofluorescence and staining

Frozen sections (5-μm thick) of rat SMGs were fixed in cold acetone for 15 min, and immunostained with primary antibody against LC-3 (1:100) overnight at 4°C, then secondary antibodies (Invitrogen, CA21206s) for 2 h at RT. Nuclei were stained with 4′, 6-diamidino-2- phenylindole (DAPI) at RT for 10 min. Fluorescence images were captured by confocal microscope (TCSSP5; Leica, Germany) and typical images were presented.

### Western blotting analysis

Total protein samples from rat SMG or SMG-C6 cell line were extracted in protein lysis buffer (in mM: 50 Tris-HCl, 5 EDTA, 5 EGTA, 150 NaCl, 40 NaF, 2.175 sodium orthovanadate, 1% TritonX-100, 0.1% SDS, 0.1% sodium deoxycholate, 0.1% aprotinin, and 1 mM phenylmethylsulfonyl fluoride, pH 7.2). Protein concentration was measured using BCA method. Equal amount of protein (25 μg) was separated by SDS/PAGE gel and transferred to PVDF membrane. The above membranes were blocked with 5% non-fat milk for 1 h, then incubated with primary antibodies overnight at 4°C. Secondary antibodies conjugated with horseradish peroxidase were incubated at RT for 2 h. Images were captured by ChemiDoc™XRS+ (Bio-Rad) with Image Lab software and quantitated by using Quantity One analysis software.

### Autophagy flux assay

SMG-C6 cells stably expressing GFP-LC3 were cultured on confocal dishes. The cells were randomly divided into three groups: control group (cultured with DMEM/F12 with 10% FBS), BTXA treatment group (cultured with control group medium and treated with 100 unit/ml, BTXA), and fasting group (DMEM/F12 without FBS). After 24 h of treatment, the cells were incubated with Lyso-Tracker Red working solution and maintained in 37°C incubator for 30 min. The cells were then washed for three times with PBS that was then replaced with fresh cell culture solution. Images were captured by using confocal microscope (TCSSP5; Leica, Germany).

### Statistical analysis

All experiments were performed in triplicate and repeated for three times using matched controls, and the data were pooled. The data were presented as mean ± S.E.M. One-way ANOVA analysis was performed to compare differences amongst groups. All statistical analyses were done using the GraphPad Prism 5.0 software under the Windows operating system. *P< 0.05 and **P< 0.01 were determined to be statistically significant.

## Results

### BTXA increased the expression and changed the distribution of autophagy-associated protein LC-3 in rat SMG

**Figure 1:**
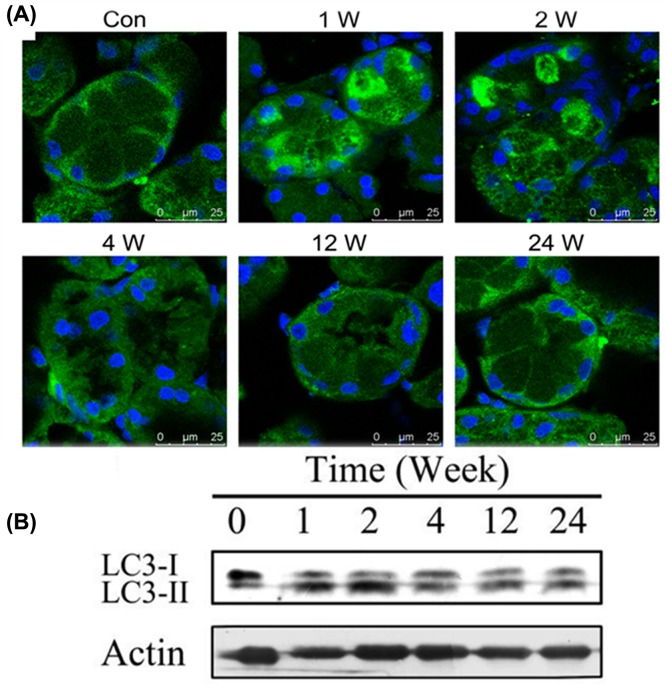
Effect of BTXA on LC3 expression and distribution in rat submandibular glands **(A)** Immunofluorescence, scale bar, 25 μm; **(B)** results of western blotting. (control group, 1 week, 2 weeks, 4 weeks, 12 weeks, and 24 weeks).

In the control group, immunofluorescence results showed that the LC-3 fluorescence was diffusely distributed in the cytoplasm of acinar cells. After BTXA injection for 1 and 2 weeks, LC-3 fluorescence was strongly spotted in the cytoplasm of acinar cells, and gradually recovered at 4 weeks. At 12 and 24 weeks, the spot-like structure disappeared and the fluorescent staining became diffusely distributed (Figure 1A). Western blotting results showed that LC3-II expression was increased at 1–2 weeks after BTXA injection and gradually declined during 4–24 weeks (Figure 1B). These results suggested that BTXA can temporarily increase the autophagosomes in rat SMG cells.

### BTXA reduced the viability of SMG-C6 cells

**Figure 2:**
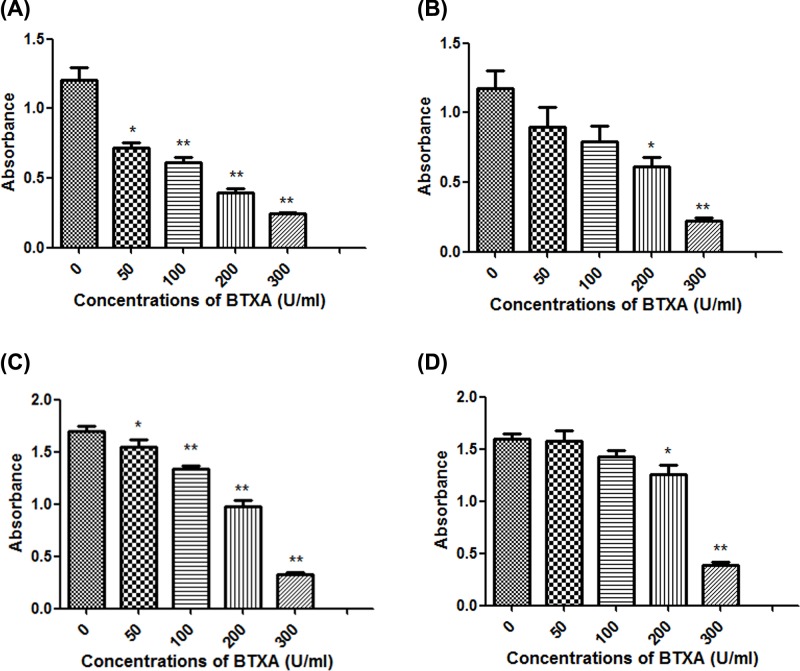
Effect of different concentrations of BTXA on SMG-C6 cells at specific times incubation time **(A)** 30 min; **(B)** 24 h; **(C)** 48 h; **(D)** 72 h. *N*=6. **P<0.01, *P<0.05.

**Figure 3:**
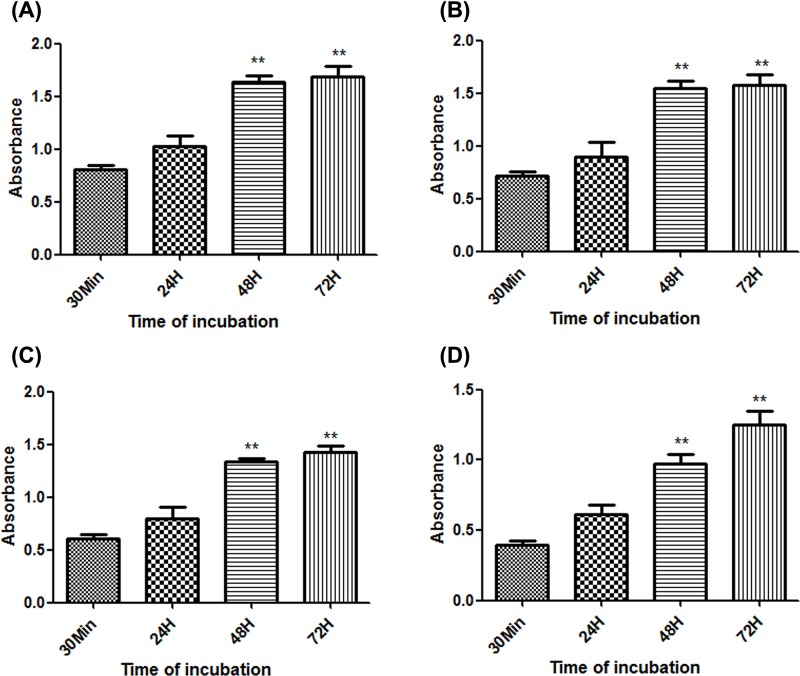
Effect of BTXA at specific concentrations on SMG-C6 cells at different incubation time Incubation concentrations were **(A)** 25 U/ml; **(B)** 50 U/ml; **(C)** 100 U/ml; **(D)** 200 U/ml. *N*=6. **P<0.01.

In order to clarify the toxic effects of BTXA on SMG-C6 cells, MTS assay was used to observe the effect of BTXA (0, 50, 100, 200, and 300 U/ml) on the viability of SMG-C6 cells for 30 min, 24, 48, and 72 h, respectively. MTS results showed that BTXA reduced the viability of SMG-C6 cells in a dose-dependent (Figure 2). In order to clarify the effect of BTXA on SMG-C6 cells proliferation, we incubated SMG-C6 cells with BTXA for different duration time and monitored their cell proliferation activities. The results showed that the inhibitory effect of BTXA on SMG-C6 cells lasted approximately 24 h (Figure 3). And when the exposure time of BTXA was exposed to 5% CO_2_ at 37°C was for 48 h, the inhibitory function of BTXA on cells disappeared and the cells started to proliferate.

### Effect of BTXA on the expression of LC3 and p62 proteins in SMG-C6 cells

**Figure 4:**
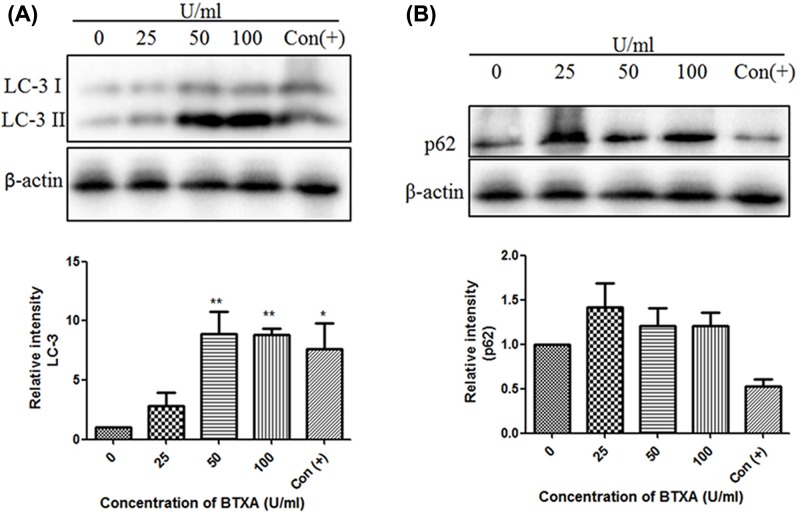
Effect of different concentrations of BTXA on LC3 and p62 protein expression Incubation concentrations of BTXA including 0 U/ml, 25 U/ml, 50 U/ml, 100 U/ml. ** (A)** LC-3, *N*=3; **(B)** p62, *N*=3. Con (+): positive control group. Mean ± S.E., **P<0.01 and *P<0.05.

To examine the effect of BTXA on LC3 and p62 proteins in SMG-C6 cells, we used different concentrations of BTXA to culture the cells for 24 h. The results showed (Figure 4) that the expression level of LC3 II was significantly increased in BTXA medium at 50 and 100 U/ml for 24 h (*n*=3, *P*<0.05). Fasting group was used as positive control. The expression level of p62 protein was relatively higher than that in the control group, but the two groups showed no statistical difference (*n*=3, *P*>0.05).

### BTXA interfered with the fusion of autophagosomes and lysosomes

**Figure 5:**
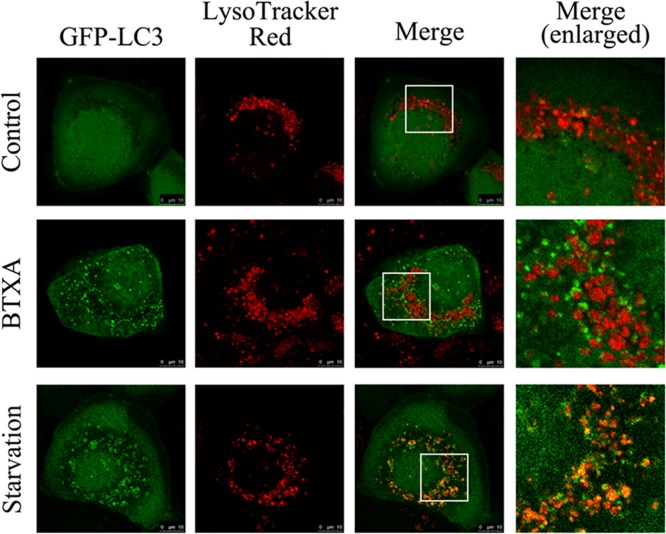
Effect of BTXA on the autophagosome and lysosome fusion in SMG-C6

In the absence of autophagy, the autophagosome marker GFP-LC3 fusion protein was diffusely distributed in the cell; when autophagy occurs, GFP-LC3 was transferred to the autophagosome membrane and appeared as bright green spots under fluorescence microscope. LysoTrackerRed is a red lysosomal fluorescent probe located on the lysosome. Under fluorescence microscopy, when autophagosomes and lysosomes did not fuse, the autophagosomes showed green fluorescent spots and lysosomes showed red fluorescent spots. After fusion, autophagosomes and lysosomes were combined. The results of immunofluorescence co-localization (Figure 5) showed that the green fluorescence was diffusely distributed in the normal control group, suggesting that fewer autophagosomes and no obvious yellow spots appeared when the two were co-localized. In the positive control group (starvation group), the green fluorescent spots significantly and the yellow spots co-localized with the lysosomes significantly increased compared with the control group. In the BTXA group, the green spots and red fluorescent spots significantly increased compared with the normal control group, indicating an increase in the number of autophagosomes and lysosomes; after co-localization, most of the green spots and red spots in the cells were separated, indicating that BTXA interfered with the fusion of autophagosomes with lysosomes.

## Discussion

Saliva is critical for maintaining an oral moisturization, digestion, and immunity. Our previous studies proved that BTXA decreased the salivation via inducing SMG cell apoptosis and changing AQP5 expression and distribution, but the hidden mechanism has not been fully illustrated. As we all know, autophagy and apoptosis are two cellular processes intertwined. Thus, we designed the present study to dig out this mechanism of autophagy.

In the present study, we first demonstrated that BTXA injection temporarily increased autophagosomes in the rat SMG cells. Further, we used rat SMG-C6 cell line as a model to explore the effect of BTXA on autophagic flux and the possible mechanisms at the cellular level.

In the cell experiments, we found that BTXA imposed its toxicity to SMG-C6 cells in a dose-dependent manner, which was consistent with the findings that high concentration of BTXA inhibited gland secretion. In order to clarify the effect of BTXA on protein expression in SMG-C6 cells, we examined the expression of autophagy-related proteins LC-3 in SMG-C6 cells. BTXA increased the conversion of LC3-I–LC3-II in a dose-dependent manner, suggesting that BTXA can increase autophagosomes in submandibular gland cells at the cellular level. However, there was no decrease in the expression of substrate protein p62 during autophagy, implying that BTXA can interfere with the degradation of autophagosomes (say, the fusion of autophagosomes and lysosomes is blocked). In order to clarify the possible causes of LC3-II increase, we performed a co-localization experiment using GFP-LC3 fusion protein and lysosomal tracer Lyso-Tracker. The results suggested that BTXA interrupted the fusion of autophagosome with lysosome.

Numerous studies in recent years have shown that abnormal autophagy is closely related to fibrosis of tissues and organs [15,16,23–25]. Pulmonary fibrosis is a dynamic process in which autophagic flow is gradually obstructed. When only inflammation and no fibrosis appear in the lung tissue, autophagy gears into an active state and autophagosomes are formed to remove the therapeutic substances; when the lung tissue turns fibrotic, the autophagic flux is blocked and the autophagy is disruppted; in this condition, autophagosomes and autophagic lysosomes formed in cells cannot be cleared [23]. Similarly, in the present study, the number of autophagosomes increased significantly at 1 and 2 weeks after BTXA injection and gradually declined at 4–24 weeks, reflecting the changes in gland structures and functions. After BTXA injection at 1 and 2 weeks, the autophagic flux in SMG cells was blocked, salivary secretion was affected and the gland structure gradually changed.

In summary, the present study found for the first time that BTXA can increase the number of autophagosomes in rat SMG and induce changes in autophagic flux in rat SMG-C6 cell line, and its potential mechanisms might be related with BTXA’s interference with autophagosme–lysosome fusion. These findings provided new insight into the mechanism of glandular secretion and structural change after BTXA injection.

## Abbreviations

BTXA: botulinum toxin type A
GFP: green fluorescent protein
LC3: light chain 3
SMG: submandibular gland
SQSTM: sequestosome
